# Association of dementia and patient outcomes among COVID-19 patients: A multi-center retrospective case-control study

**DOI:** 10.3389/fmed.2022.1050747

**Published:** 2022-11-07

**Authors:** Pratikkumar H. Vekaria, Areej Syed, Jeffrey Anderson, Brendon Cornett, Amine Bourbia, Michael G. Flynn, Rahul Kashyap, Asif R. Shah

**Affiliations:** ^1^Prisma Health-Upstate, Greenville, SC, United States; ^2^Redmond Regional Medical Center, Advent Health, Rome, GA, United States; ^3^HCA Healthcare, Nashville, TN, United States; ^4^Mayo Clinic, Rochester, MN, United States

**Keywords:** dementia, COVID-19, mortality, baseline characteristics, intensive care unit (ICU)

## Abstract

**Background:**

We conducted a retrospective cohort study on COVID-19 patients with and without dementia by extracting data from the HCA Healthcare Enterprise Data Warehouse between January-September 2020.

**Aims:**

To describe the role of patients' baseline characteristics specifically dementia in determining overall health outcomes in COVID-19 patients.

**Methods:**

We grouped in-patients who had ICD-10 codes for dementia (DM) with age and gender-matched (1:2) patients without dementia (ND). Our primary outcome variables were in-hospital mortality, length of stay, Intensive Care Unit (ICU) admission, ICU-free days, mechanical ventilation (MV) use, MV-free days and 90-day re-admission.

**Results:**

Matching provided similar age and sex in DM and ND groups. BMI (median, 25.8 vs. 27.6) and proportion of patients who had smoked (23.3 vs. 31.3%) were lower in DM than in ND patients. The median (IQR) Elixhauser Comorbidity Index was higher in dementia patients 7 (5–10) vs. 5 (3–7, *p* < 0.01). Higher mortality was observed in DM group (30.8%) vs. ND group (26.4%, *p* < 0.01) as an unadjusted univariate analysis. The 90-day readmission was not different (32.1 vs. 31.8%, *p* = 0.8). In logistic regression analysis, the odds of dying were not different between patients in DM and ND groups (OR = 1.0; 95% CI 0.86–1.17), but the odds of ICU admissions were significantly lower for dementia patients (OR = 0.58, 95% CI 0.51–0.66).

**Conclusions:**

Our data showed that COVID-19 patients with dementia did not fare substantially worse, but in fact, fared better when certain metrics were considered.

## Introduction

The novel Coronavirus disease-2019 (COVID-19), caused by severe acute respiratory syndrome coronavirus 2, is responsible for the current global pandemic (1). As of September 5, 2022, there were a total of >94 million cases with >1 million deaths reported in the USA (2). We have several treatments and preventative options, including vaccines, available now, but patients' baseline characteristics (3) also play a major role in the overall health outcomes. Patients' characteristics such as age, hypertension, diabetes, renal failure, obesity (4), high SOFA score, and elevated D-dimer levels can lead to a poor outcome for those with COVID-19 infections (5, 6). However, there are other conditions that may help patients recover faster from this disease. For example, a retrospective study conducted in Denmark showed that people with blood type O and Rh-negative were less susceptible and possessed natural protection against COVID-19 infection (7, 8). The elderly population has been disproportionately and negatively impacted by the illness and dementia is one of the unexplored characteristics. There is paucity of data denoting association between dementia and outcomes among COVID-19 patients.

Dementia is a condition in which impairment of cognitive functions—thinking, reasoning, memory, and behavioral abilities occur that can negatively affect the patient's life and daily activities (8). There are no strong supporting data available to date showing the association between dementia and COVID-19 outcomes. It is therefore essential to collect more information on this topic by conducting a cohort study and doing an appropriate systematic analysis of the data. We conducted a multi-center retrospective study to determine if an association exists between dementia and COVID-19 outcomes in a larger patient population. We assessed several laboratory and clinical variables that might help explain the relationship should it exist.

The aim of the study is to describe the role of dementia in determining overall health outcomes in COVID-19 patients. We hypothesized that there were no differences in patient outcomes for patients with COVID-19 infection (60+ years of age), with and without a diagnosis of dementia.

## Materials and methods

We conducted a retrospective case-control study by extracting data from the HCA Healthcare Enterprise Data Warehouse (EDW), which included data from 160 hospitals at the time it was extracted, with all the cases accrued between January 1, 2020, and September 30, 2020. We grouped in-patients who had ICD-10 codes for dementia (F03.90/F01/G31.09) with age- and gender-matched (1:2) patients without a dementia diagnosis. Our primary outcome variables were in-hospital mortality, length of hospital stay (LOS), Intensive Care Unit (ICU) admissions rate, ICU-free days, mechanical ventilation (MV) use, MV-free days, and 90-day re-admission.

Independent group variables were compared using a Mann-Whitney U (Wilcoxon Rank Sum Test) or a G-Test (Likelihood Ratio Chi-Squared Test) for continuous and categorical variables, respectively. Logistic regression or negative binomial regression models were created, as appropriate, for patient mortality, ICU admission, mechanical ventilator (required), length of hospital admission, and length of ventilator use. Selected covariates were included in each model to adjust for the main predictor variable, dementia diagnosis. The Elixhauser Comorbidity Index (ECI) was included, by taking into account chronic kidney disease, diabetes, COPD, hypertension, heart failure, liver disease, cerebral infarction, atherosclerotic and malignancy, to predict disease severity in both dementia and non-dementia groups. The study was not powered for the inclusion of covariates and these relationships will be interpreted with caution. An alpha level of 0.05 was selected a priori as the level of significance.

This study was reviewed by Institutional Review Board (IRB) and they waived the need for approval on 3/30/2021. The procedures were followed in accordance with the ethical standards of the responsible committee on human experimentation (institutional or regional) and with the Helsinki Declaration of 1975. Our internal reference number for this determination was 2021-291.

### Inclusion criteria

As mentioned above, we conducted this study by extracting data from the HCA Healthcare Enterprise Data Warehouse (EDW) between January 1, 2020, and September 30, 2020. Considering the retrospective nature of the study, investigators' blinding was not possible, and data were measured as objectively as possible. The patients were included in this study based on three criteria: (1) Patients' age ≥60 but ≤89 years, (2) Patients with COVID-19 infection, (3) Patients with and without dementia.

### Exclusion criteria

We excluded patients as per the following criteria: 1. Patients <60 years of age, or >89 years of age, 2. BMI <15 or >75, 3. Patients with pre-existing neurodevelopmental, psychiatric disorders, or 4. Baseline characteristics data were not available.

## Results

**Figure 1:**
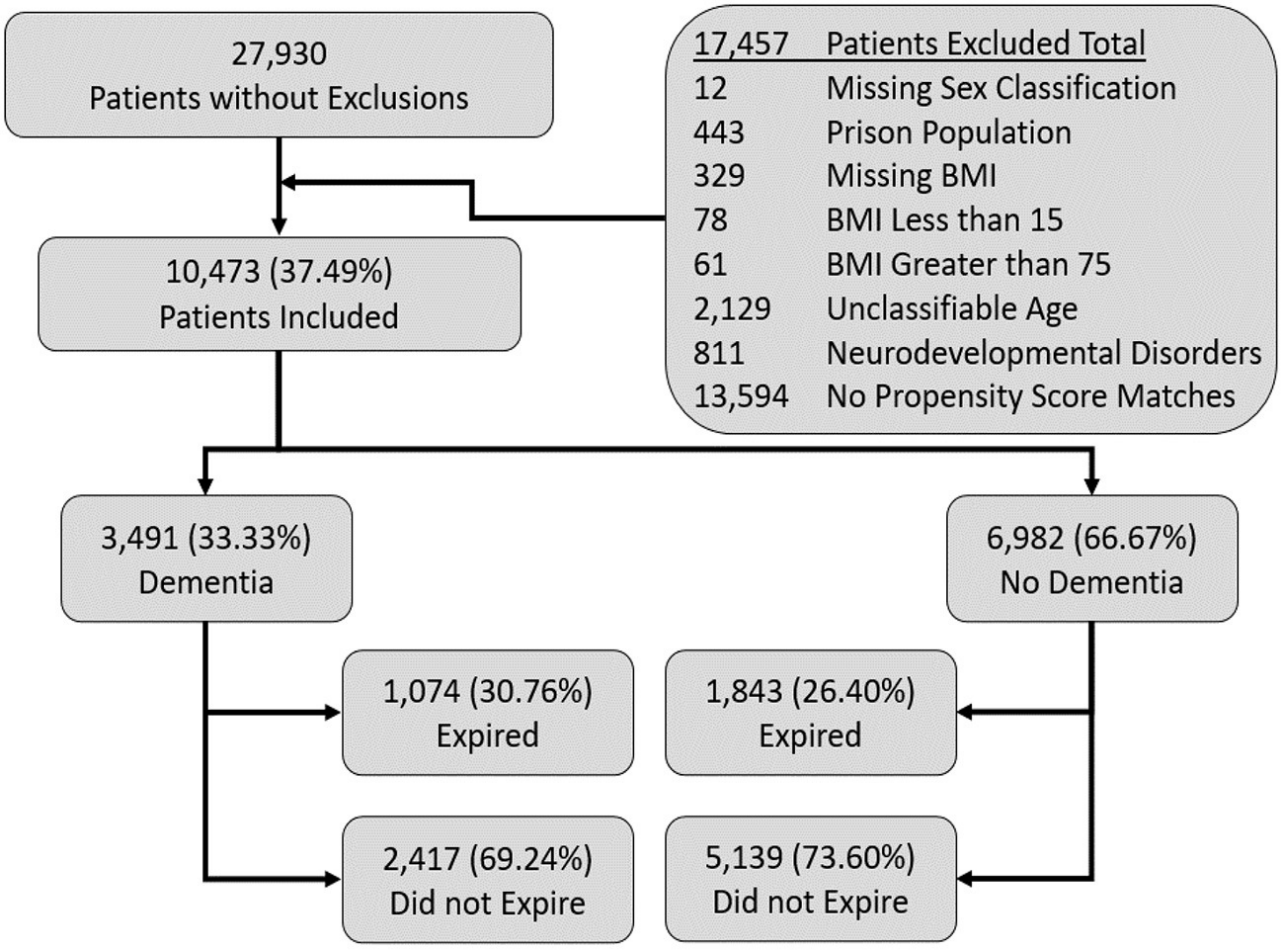
Flow diagram of included patients in the study.

We screened a total of 27, 930 patients from which we included 10,473 patients per our inclusion and exclusion criteria. Among the 10,473 included patients, 3,491 patients had dementia (DM) and 6,982 were included, after matching, who did not have dementia (ND) as mentioned in the Figure 1.

Table 1 shows the demographic data for patients included in this study. As expected, matching elicited similar age (median age: 79 vs. 79) and sex (F vs. M, 50.1 vs. 49.9%) in DM and ND groups; however, BMI (25.8 vs. 27.6) and smoking exposure (23.3 vs. 31.3%) were lower in DM vs. ND, respectively. In addition, the DM group had a somewhat higher proportion of African-American patients than the ND group.

**Table 1:** Demographic variables included in the study such as age, sex, race, BMI and smoking status.

**Demographic variables**	**Dementia (*N* = 3,491)**	**No dementia (*N* = 6,982)**	***p*-value**	**All patients (*N* = 10,473)**
Age				
Median (IQR)	79 (73–83)	79 (73–82)	0.80*	79 (73–83)
Range	60–89	60–89		60–89
Sex			0.06^‡^	
Female	1,749 (50.10%)	3,361 (48.14%)		5,110 (48.79%)
Male	1,742 (49.90%)	3,621 (51.86%)		5,363 (51.21%)
Race			<0.01^‡^	
African American	738 (21.14%)	1,185 (16.97%)		1,923 (18.36%)
Hispanic	2 (0.06%)	5 (0.07%)		7 (0.07%)
Multiracial/other	472 (13.52%)	1,301 (18.63%)		1,773 (16.93%)
Caucasian	2,279 (65.28%)	4,491 (64.32%)		6,770 (64.64%)
BMI				
Median (IQR)	25.83 (22.51–29.63)	27.61 (24.29–32.07)	<0.01*	27.11 (23.72–31.31)
Range	15.00–67.27	15.02–73.65		15.00–73.65
BMI Groups			<0.01^‡^	
<20	361 (10.34%)	348 (4.98%)		709 (6.77%)
20–24.9	1,127 (32.28%)	1,719 (24.62%)		2,846 (27.17%)
25–29.9	1,194 (34.20%)	2,451 (35.10%)		3,645 (34.80%)
30–34.5	513 (14.69%)	1,374 (19.68%)		1,887 (18.02%)
≥35	296 (8.48%)	1,090 (15.61%)		1,386 (13.23%)
Ever smoked	812 (23.26%)	2,189 (31.35%)	<0.01^‡^	3,001 (28.65%)

*Mann-Whitney U test (Wilcoxon Rank Sum Test),^‡^G-Test (Likelihood Ratio Chi-Squared Test).

IQR, Interquar tile Range; BMI, Body Mass Index.

Higher mortality was observed in the DM group (30.8%) vs. ND group (26.4%, *p* < 0.01) in the unadjusted univariate analysis (Table 2). However, a dementia diagnosis was not a significant predictor of mortality in our logistic regression [odds ratio (OR), 1.00; 95% CI, 0.86–1.17] when controlling for a range of covariates (Table 3). As shown in the summary of univariate analyses in Table 2, ICU admission (30.7 vs. 34%, *p* < 0.01) and mechanical ventilation use (9.5 vs. 14.5%, *p* < 0.01) were significantly lower in the DM group than in the ND group, and ICU-free days (6 vs. 5, *p* < 0.01), and ventilator-free days (8 vs. 6, *p* < 0.01) were significantly higher, respectively. There was no difference in the 90-day readmission between DM and ND groups (32.1 vs. 31.8%, Table 2). A higher proportion of dementia patients had DNR orders (41.2 vs. 27.7%, *p* < 0.01, Table 2).

**Table 2:** Primary outcomes for all patients, patients with dementia, and patients with no dementia.

**Primary outcomes with important vitals**	**Dementia (*N* = 3,491)**	**No dementia (*N* = 6,982)**	***p*-value**	**All patients (*N* = 10,473)**
In hospital mortality	1,074 (30.76%)	1,843 (26.40%)	<0.01^‡^	2,917 (27.85%)
Do not resuscitate documented	1,439 (41.22%)	1,937 (27.74%)	<0.01^‡^	3,376 (32.24%)
Length of stay–days				
Median (IQR)	8 (4–14)	7 (4–13)	<0.01*	7 (4–13)
Range	1–132	1–99		1–132
Readmitted within 90 days	1,120 (32.08%)	2,224 (31.85%)	0.81^‡^	3,344 (31.93%)
ICU admission	1,070 (30.65%)	2,373 (33.99%)	<0.01^‡^	3,443 (32.88%)
Mechanical ventilation used	332 (9.51%)	1,011 (14.48%)	<0.01^‡^	1,343 (12.82%)
Days not in ICU				
Median (IQR)	6.04 (3.00–12.00)	5.00 (2.00–9.00)	<0.01*	5.00 (2.25–10.00)
Range	0.00–132.00	0.00–90.13		0.00–132.00
Days not on ventilator				
Median (IQR)	8 (4–13)	6 (3–11)	<0.01*	6 (3–12)
Range	0–132	0–93		0–132
Low SBP (<90)	414 (11.86%)	583 (8.35%)	<0.01^‡^	997 (9.52%)
High Respiratory Rate (≥ 30)	689 (19.74%)	1,420 (20.34%)	0.47^‡^	2,109 (20.14%)
High Pulse Rate (> 125)	336 (9.62%)	569 (8.15%)	0.01^‡^	905 (8.64%)

*Mann-Whitney U test (Wilcoxon Rank Sum Test),^‡^G-Test (Likelihood Ratio Chi-Squared Test).

IQR, Interquartile range; ICU, Intensive Care Unit; SBP, Systolic Blood Pressure.

**Table 3:** Logistic regression for patient mortality by dementia diagnosis.

**Variable**	**Odds ratio**	**95% wald confidence interval**		***p*-value**
Main predictor
Dementia diagnosis	1.00	0.86	1.17	0.99
Covariates
Length of stay (per day)	0.97	0.95	0.99	<0.01*
Age (per year)	1.03	1.02	1.05	<0.01*
Female vs. male	0.70	0.60	0.81	<0.01*
Chronic kidney disease diagnosis	1.15	0.97	1.37	0.10
COPD diagnosis	1.00	0.84	1.19	0.99
Diabetes diagnosis	0.91	0.78	1.07	0.25
Hypertension diagnosis	0.73	0.58	0.92	<0.01*
Infarction diagnosis	0.97	0.62	1.51	0.89
Liver disease diagnosis	1.12	0.79	1.59	0.52
Arterial plaque diagnoses	1.06	0.91	1.24	0.45
Any tumors present	0.76	0.20	2.94	0.69
Elixhauser comorbidity index (per unit)	1.06	1.03	1.10	<0.01*
BMI <20 vs. BMI between 20 and 24.9	1.12	0.85	1.46	0.31
BMI between 25 and 29.9 vs. BMI between 20 and 24.9	1.03	0.86	1.23	0.72
BMI between 30 and 34.5 vs. BMI between 20 and 24.9	1.01	0.81	1.26	0.97
BMI >35 vs. BMI between 20 and 24.9	0.89	0.68	1.15	0.20
Admitted to intensive care unit (ICU)	3.48	2.95	4.11	<0.01*
Smoker	0.82	0.70	0.97	0.02
Time not on ventilator (per day)	0.99	0.97	1.02	0.58
Systolic BP ever below 90	1.01	0.80	1.27	0.94
Respiration rate ever above 30	1.38	1.16	1.65	<0.01*
Pulse rate ever above 125	1.11	0.88	1.41	0.37
Mechanical ventilator used	6.11	4.48	8.33	<0.01*
Documented do not resuscitate (DNR)	13.97	12.03	16.22	<0.01*

*Mann-Whitney U test (Wilcoxon Rank Sum Test).

BMI, Body Mass Index; COPD, Chronic Obstructive Pulmonary Disease.

The Elixhauser Comorbidity Index (ECI) was significantly higher in the DM group than in the ND group (7 vs. *p* < 0.01) when dementia was included in the ECI score (Table 4). The most frequent comorbidities were hypertension (DM, 90.29 vs. ND, 87.15%), diabetes (52.22 vs. 49.41%), and chronic kidney disease (36.92 vs. 32.28%), all significantly higher (*p* < 0.01) in the DM patient group (Table 4).

**Table 4:** The distribution of co-morbidities among the two groups under study; dementia vs. no dementia.

**Co-morbidities**	**Dementia (*N* = 3,491)**	**No dementia (*N* = 6,982)**	***p*-value**	**All patients (*N* = 10,473)**
Elixhauser comorbidity index with dementia				
Median (IQR)	7 (5–10)	5 (3–7)	<0.01*	6 (4–8)
Range	2–25	0–27		0–27
Chronic kidney disease	1,289 (36.92%)	2,254 (32.28%)	<0.01^‡^	3,543 (33.83%)
COPD	874 (25.04%)	1,669 (23.90%)	0.20^‡^	2,543 (24.28%)
Diabetes	1,823 (52.22%)	3,450 (49.41%)	<0.01^‡^	5,273 (50.35%)
Heart failure	939 (26.90%)	1,815 (26.00%)	0.32^‡^	2,754 (26.30%)
Hypertension	3,152 (90.29%)	6,085 (87.15%)	<0.01^‡^	9,237 (88.20%)
Cerebral infarction	94 (2.69%)	145 (2.08%)	0.05^‡^	239 (2.28%)
Liver disease	134 (3.84%)	289 (4.14%)	0.46^‡^	423 (4.04%)
Atherosclerosis and similar conditions	1,227 (35.15%)	2,344 (33.57%)	0.11^‡^	3,571 (34.10%)
Malignant tumors	7 (0.20%)	13 (0.19%)	0.88^‡^	20 (0.19%)

*Mann-Whitney U test (Wilcoxon Rank Sum Test),^‡^G-Test (Likelihood Ratio Chi-Squared Test).

IQR, Interquartile Range; COPD, Chronic Obstructive Pulmonary Disease.

As mentioned above, after correction for covariates in the regression analysis, a dementia diagnosis was not an independent predictor of patient mortality. However, patients with a dementia diagnosis had significantly lower odds (OR = 0.58; 95% CI 0.51–0.66, *p* < 0.01) of an ICU admission (Table 5) and lower odds for the need of mechanical ventilation (OR = 0.53; 95% CI 0.43–0.65, *p* < 0.01) than patients without a dementia diagnosis (Supplementary Table 1).

**Table 5:** Logistic regression for ICU admission by dementia diagnosis.

**Variable**	**Odds ratio**	**95% confidence interval**		***p*-value**
Main predictor
Dementia diagnosis	0.58	0.51	0.66	<0.01
Covariates
Age (per year)	0.98	0.97	0.99	<0.01
BMI <20 vs. BMI between 20 and 24.9	0.98	0.79	1.21	0.85
BMI between 25 and 29.9 VS. BMI between 20 and 24.9	1.07	0.92	1.24	0.19
BMI between 30 and 34.5 vs. BMI between 20 and 24.9	1.06	0.88	1.27	0.34
BMI >35 vs. BMI between 20 and 24.9	0.88	0.69	1.13	0.19
Female vs. male	0.88	0.78	1.00	0.04
Chronic kidney disease diagnosis	0.88	0.76	1.02	0.08
COPD diagnosis	0.83	0.72	0.97	0.02
Diabetes diagnosis	1.00	0.88	1.15	0.96
Hypertension diagnosis	1.17	0.95	1.44	0.14
Infarction diagnosis	1.37	0.94	1.98	0.10
Liver disease diagnosis	1.10	0.82	1.48	0.52
Arterial plaque diagnoses	0.94	0.82	1.07	0.33
Any tumors present	0.71	0.19	2.74	0.62
Elixhauser comorbidity index (per unit)	1.11	1.08	1.13	<0.01
Expired	5.12	4.39	5.97	<0.01
Smoker	1.28	1.12	1.47	<0.01
Time not on ventilator (per day)	1.05	1.04	1.05	<0.01
SBP ever below 90	2.62	2.16	3.18	<0.01
Respiration rate ever above 30	3.85	3.33	4.44	<0.01
Pulse rate ever above 125	1.19	0.97	1.47	0.09
Mechanical ventilator used	1.07	0.92	1.24	0.41

BMI, Body Mass Index; COPD, Chronic Obstructive Pulmonary Disease; SBP, Systolic Blood Pressure.

A dementia diagnosis was not a significant main predictor of the length of hospital admission with an incidence rate ratio (IRR) of 1.00 (95% CI 0.98–1.02, Supplementary Table 2). In contrast, a dementia diagnosis was a significant (*p* < 0.01) main predictor of the length of ventilator use (IRR = 0.51; 95% CI 0.43–0.61, Supplementary Table 3).

Among the covariates, few had meaningful effect sizes and those with modest to strong effect sizes such as admitted to ICU (OR = 3.48; 95% CI 2.95–4.11) or documented Do Not Resuscitate (DNR) (OR = 13.97; 95% CI 12.03–16.22) in the patient mortality model (Table 3), did not contribute substantially to data interpretation. However, respiration rate over 30 (OR = 3.85; 95% CI 3.33–4.44, Table 5), systolic blood pressure (SBP) below 90 mmHg (OR = 2.86; 95% CI 2.23–3.68) and pulse rate (PR) above 125 (OR = 1.49; 95% CI 1.12–1.97, Supplementary Table 1) yielded moderate effects predictive of patient's ICU admission and mechanical ventilator use, respectively. Similarly, SBP below 90 mmHg (IRR = 2.05; 95% CI 1.63–2.59), pulse rate above 125 (IRR = 1.95; 95% CI 1.63–2.32, Supplementary Table 3) were significantly related to length of ventilator use.

## Discussion

In a 1:2 matched Dementia and Non-Dementia groups, we found higher unadjusted in-hospital mortality in the dementia group, but co-morbidity-adjusted logistic regression analysis did not reveal a significant odds for higher mortality. Dementia was a significant main predictor of lower odds of ICU admissions, mechanical ventilator use, and shorter length of ventilator use.

A meta-analysis of 24 studies involving 46,391 patients showed that higher mortality was observed in patients with dementia from the COVID-19 infection [RR 2.62] (9). Most of the studies in this analysis had fewer than 5–10% dementia patients. The meta-analysis also did not control for age and co-morbidities, while our model with dementia diagnosis as the main predictor controlled for a range of demographic factors and co-morbidities. Pisaturo et al. suggested that in this meta-analysis the increased mortality in the patients with dementia could be related to the presence of multiple co-pathologies and the negative impact of age (10). In the UK, a large community cohort study was conducted on COVID-19 patients, and it showed that patients with dementia were at high risk for COVID-19 hospitalization (OR = 3.50; 1.93–6.34) and at increased risk for COVID-19 associated death (OR = 7.30; 3.28–16.21) (11). This community cohort study had only 14 patients who were positive for dementia, and the statistical power was far lower compared to the rest of the UK Biobank sample (11).

A retrospective study conducted in Wuhan, China showed that baseline characteristics such as chronic heart problems or advanced age can lead to high mortality in the patients with COVID-19 (6). A meta-analysis of 217 observational studies from 26 countries involving 624,986 patients indicated that patients with chronic diseases, including dementia, were more likely to experience ICU admission, severe illness, and higher mortality (12). Several studies lend support to the hypothesis that mortality is higher in those with dementia as mentioned above; however, not enough data are available to conclude lower morbidity and degree of critical illness in the patient population in question. Our study supports the findings that dementia patients are relatively sicker (high ECI score: 7 vs. 5) compared to non-dementia patients. Despite these findings, ICU admission and MV-use rate were lower in the dementia group.

Pathophysiologically, angiotensin-converting enzyme 2 (ACE-2) receptor, the cellular receptor for the COVID-19, present on the brain and glial tissue makes the CNS (central nervous system) a likely target for this virus (13). The Alzheimer's Association International Conference (AAIC) 2021 in Denver indicated the connections between COVID-19 and cognitive deficits such as the acceleration of Alzheimer's disease (14). In this cohort study, patients with dementia had higher unadjusted mortality but had fewer ICU admissions, mechanical ventilation use, more ICU-free days, and ventilator-free days. It is possible that patients with dementia are considered to have a shorter life expectancy or poor quality of life, and hence, are less prone to receiving aggressive ICU-level care. On the other hand, there may be pathophysiological effects in the patients with dementia, currently poorly understood, that help them recover from COVID 19 infection sooner, even though overall mortality is believed to be worse. It is postulated that the patients with dementia are less aware of or unable to comprehend the disease severity, disease-associated mortality, and media hype. Therefore, patients with dementia might have lower stress compared to patients without dementia. Elderly patients with dementia are prone to have chronic inflammatory changes which can negatively affect the acquired immune system (15). In addition to that, stress has a substantial impact on the immune system. Catecholamine and suppressor T-cells levels could be increased with the stress which could further suppress immunity (16). The lack of this effect in the DM group could have possibly favored better outcomes, as we are observed, in our study. More studies need to be done to confirm the causal relationship.

Our study had several strengths. We had a large sample size which provided higher statistical power. Furthermore, the racial distribution in our study groups was similar to the general population. Our logistic regression results were also adjusted for multiple covariates such as BMI and comorbidities which could significantly otherwise confound the mortality outcomes. Furthermore, our study timing could have played a role in the overall outcomes. Our study includes a patient population from the early stages of the pandemic. At that time, patients didn't have complete understanding of this disease condition and it could have favored the outcomes specifically for the DM group due to psychosocial factors such as low-stress levels as mentioned above.

Our study also has some limitations. The work was retrospective in nature, thus unknown confounders couldn't be controlled. Another limitation is that the data were pulled based on ICD-10 codes, so it has inherent weaknesses of such automation. We also could not differentiate the stages of dementia based on ICD-10 codes. Dementia patients are also prone to have more DNR, hospice care, or comfort care code status, as shown in the results, which could influence the clinical decision-making. Dementia patients are less likely to receive life-prolonging care such as mechanical ventilation, ICU care. Hospice care/palliative care has an impact on mortality measurement (17), and it may have affected our outcomes in the dementia group. Patients with hospice or comfort care were included under DNR code status and we were unable to extract the data on the total number of dementia patients who opted in for hospice or comfort care. Therefore, it might be difficult to assess if the mortality in dementia patients was inflated due to these factors. Furthermore, recent data showed that secondary bacterial infection could affect mortality and other outcomes in ICU and non-ICU patients, and bacteremia might be more frequent in dementia patients (18). We did not have data available on this measure. Moreover, our study data is limited to the first wave of the COVID-19 pandemic, and we had limited resources which may affect the clinical outcomes.

There is a need for pooling the data from hospitals across the country and internationally to validate these results from a large hospital system (19). There may be variations in hospital practice, which need to be taken into account while interpreting results from our study (20).

## Conclusion

Our study findings are contrary to several studies in the literature in which poorer outcomes for patients with dementia were reported. We found indifferent or favorable outcomes compared to non-dementia patients with COVID-19 infection. It is reasonable to extrapolate that there are potential positive morbidity effects in patients with dementia who are infected with COVID 19 virus. It is also possible that these positive outcomes may be mediated by other unmeasurable factors such as more medical attention due to their dementia condition with respect to age-matched non-dementia patients.

## Data availability statement

The original contributions presented in the study are included in the article/Supplementary material, further inquiries can be directed to the corresponding author/s.

## Ethics statement

The studies involving human participants were reviewed and approved by HCA Healthcare-Institutional Review Board (IRB) and they waived the need for approval on 3/30/2021. Written informed consent for participation was not required for this study in accordance with the national legislation and the institutional requirements.

## Author contributions

PV contributed to the study concept and design, drafting of the article, analysis and interpretation of data, administrative and critical revision of the article for important intellectual content, had full access to all the data, and he was responsible for the manuscript submission for publication. ASy helped in drafting the article, analysis and interpretation of data. JA and AB helped in critical revision of the article for important intellectual content. BC helped in acquisition of data, technical and material support, and analysis and interpretation of data. MF and RK helped in study concept and design, analysis and interpretation of data, technical and material support, and critical revision of the article for important intellectual content. ASh helped in study concept and design, administrative, analysis and interpretation of data, and critical revision of the article for important intellectual content. All authors contributed to the article and approved the submitted version.

## Funding

This research was supported (in whole or in part) by HCA Healthcare and/or an HCA Healthcare affiliated entity. Prisma Health-Upstate is partly supporting the publication expenses for this paper.

## Conflict of interest

Authors ASy, AB, ASh, and PV were employed by Advent Health. Authors JA, BC, MF, and RK were employed by HCA Healthcare.

## Publisher's note

All claims expressed in this article are solely those of the authors and do not necessarily represent those of their affiliated organizations, or those of the publisher, the editors and the reviewers. Any product that may be evaluated in this article, or claim that may be made by its manufacturer, is not guaranteed or endorsed by the publisher.

## Author disclaimer

The views expressed in this publication represent those of the author(s) and do not necessarily represent the official views of HCA Healthcare or any of its affiliated entities.
