# Supplementary material for: Association of dementia and patient outcomes among COVID-19 patients: A multi-center retrospective case-control study
Source: Front Med (Lausanne). 2022 Nov 7;9:1050747. doi: 10.3389/fmed.2022.1050747 (PMC9676493; doi:10.3389/fmed.2022.1050747)
Supplement: Supplementary file 1 [file Table_1.pdf]

Supplementary Table 1. Logistic Regression for Mechanical Ventilator Use by Dementia Diagnosis

Variable	Odds Ratio	95% Confidence Interval		p-Value
Main Predictor				
Dementia Diagnosis	0.53	0.43	0.65	<0.01
Covariates				
Length of Stay (per day)	1.15	1.14	1.17	<0.01
Chronic Kidney Disease Diagnosis	0.99	0.78	1.24	0.90
COPD Diagnosis	0.85	0.66	1.08	0.17
Diabetes Diagnosis	1.03	0.83	1.28	0.79
Hypertension Diagnosis	1.07	0.74	1.53	0.73
Infarction Diagnosis	1.05	0.61	1.78	0.87
Liver Disease Diagnosis	1.02	0.66	1.59	0.92
Arterial Plaque Diagnoses	0.90	0.73	1.11	0.31
Any Tumors Present	0.79	0.11	5.90	0.82
Age (per year)	0.97	0.96	0.99	<0.01
African American VS. Caucasian	1.45	1.11	1.89	0.43
Hispanic VS. Caucasian	0.46	0.02	10.79	0.49
Multiracial/Other VS. Caucasian	1.82	1.41	2.35	0.18
Elixhauser Comorbidity Index (per unit)	1.06	1.02	1.10	<0.01
Admitted to ICU	4.91	3.67	6.57	<0.01
BMI less than 20 VS. BMI between 20 and 24.9	0.79	0.51	1.20	0.02
BMI between 25 and 29.9 VS. BMI between 20 and 24.9	1.19	0.92	1.54	0.70
BMI between 30 and 34.5 VS. BMI between 20 and 24.9	1.49	1.10	2.01	0.01

Supplementary Table 1. Logistic Regression for Mechanical Ventilator Use by Dementia Diagnosis

Variable	Odds Ratio	95% Confidence Interval		p-Value
BMI greater than 35 VS. BMI between 20 and 24.9	1.43	1.02	2.02	0.08
Expired	6.88	5.31	8.91	<0.01
Time not in ICU (per day)	0.90	0.88	0.91	<0.01
SBP ever below 90	2.86	2.23	3.68	<0.01
Pulse Rate ever above 125	1.49	1.12	1.97	0.01
Smoker	1.11	0.89	1.39	0.35
Documented DNR	1.06	0.83	1.35	0.64

Notes: COPD = Chronic Obstructive Pulmonary Disease; ICU = Intensive Care Unit; BMI = Body Mass Index; SBP = Systolic Blood Pressure; DNR = Do Not Resuscitate

Supplementary Table 2. Negative Binomial Regression for Length of Hospital Admission by Dementia Diagnosis

Variable	Incidence Rate Ratio	95% Confidence Interval		p-Value
Main Predictor				
Dementia Diagnosis	1.00	0.98	1.02	0.82
Covariates				
Age (per year)	1.00	1.00	1.00	0.07
BMI less than 20 VS. BMI between 20 and 24.9	0.98	0.94	1.01	0.18
BMI between 25 and 29.9 VS. BMI between 20 and 24.9	1.01	0.99	1.03	0.40
BMI between 30 and 34.5 VS. BMI between 20 and 24.9	1.00	0.97	1.03	0.99
BMI greater than 35 VS. BMI between 20 and 24.9	1.00	0.97	1.03	0.88
Chronic Kidney Disease Diagnosis	0.97	0.95	0.99	<0.01
COPD Diagnosis	0.99	0.97	1.01	0.24
Diabetes Diagnosis	0.99	0.97	1.01	0.16
Hypertension Diagnosis	1.01	0.98	1.04	0.54
Infarction Diagnosis	1.00	0.95	1.06	0.95
Liver Disease Diagnosis	1.05	1.01	1.10	0.02
Arterial Plaque Diagnoses	0.98	0.96	1.00	0.03
Any Tumors Present	1.07	0.87	1.30	0.53
Elixhauser Comorbidity Index (per unit)	1.02	1.01	1.02	<0.01
Admitted to ICU	1.19	1.16	1.22	<0.01
Expired	0.99	0.97	1.02	0.59
Time not on Ventilator (per day)	1.06	1.05	1.06	<0.01
Time not in ICU (per day)	1.01	1.01	1.02	<0.01
SBP ever below 90	0.92	0.89	0.95	<0.01

Supplementary Table 2. Negative Binomial Regression for Length of Hospital Admission by Dementia Diagnosis

Variable	Incidence Rate Ratio	95% Confidence Interval		p-Value
Mechanical Ventilator Used	2.04	1.98	2.10	<0.01
Smoker	0.99	0.97	1.01	0.57
Documented DNR	1.04	1.01	1.06	<0.01

Notes: BMI = Body Mass Index; COPD = Chronic Obstructive Pulmonary Disease; ICU = Intensive Care Unit; SBP = Systolic Blood Pressure; DNR = Do Not Resuscitate

Supplementary Table 3. Negative Binomial Regression for Length of Ventilator Use by Dementia Diagnosis

Variable	Incidence Rate Ratio	95% Confidence Interval		p-Val-ue
Main Predictor				
Dementia Diagnosis	0.51	0.43	0.61	<0.01
Covariates				
Age (per year)	0.96	0.95	0.97	<0.01
African American VS. Caucasian	1.53	1.24	1.89	<0.01
Hispanic VS. Caucasian	0.15	0.01	1.95	0.15
Multiracial/Other VS. Caucasian	1.67	1.35	2.07	<0.01
BMI less than 20 VS. BMI between 20 and 24.9	0.45	0.32	0.65	<0.01
BMI between 25 and 29.9 VS. BMI between 20 and 24.9	0.94	0.77	1.16	0.59
BMI between 30 and 34.5 VS. BMI between 20 and 24.9	1.14	0.89	1.45	0.30
BMI greater than 35 VS. BMI between 20 and 24.9	0.98	0.75	1.30	0.91
Chronic Kidney Disease Diagnosis	0.69	0.57	0.84	<0.01
COPD Diagnosis	0.61	0.50	0.74	<0.01
Diabetes Diagnosis	0.96	0.80	1.14	0.61
Hypertension Diagnosis	0.95	0.72	1.26	0.72
Infarction Diagnosis	1.59	0.97	2.58	0.06
Liver Disease Diagnosis	1.97	1.39	2.81	<0.01
Arterial Plaque Diagnoses	0.83	0.70	0.98	0.03
Any Tumors Present	0.55	0.11	2.75	0.47
Elixhauser Comorbidity Index (per unit)	1.13	1.09	1.17	<0.01
Admitted to ICU	34.68	28.53	42.15	<0.01
Expired	5.24	4.35	6.31	<0.01

Supplementary Table 3. Negative Binomial Regression for Length of Ventilator Use by Dementia Diagnosis

Variable	Incidence Rate Ratio	95% Confidence Interval		p-Value
Time not in ICU (per day)	1.09	1.08	1.10	<0.01
SBP ever below 90	2.05	1.63	2.59	<0.01
Pulse Rate ever above 125	1.95	1.63	2.32	<0.01
Smoker	1.08	0.90	1.30	0.39
Documented DNR	1.28	1.06	1.53	0.01

Notes: BMI = Body Mass Index; COPD = Chronic Obstructive Pulmonary Disease; ICU = Intensive Care Unit; SBP = Systolic Blood Pressure; DNR = Do Not Resuscitate
